# Supplementary material for: Seasonal switching of integrated leaf senescence controls in an evergreen perennial Arabidopsis
Source: Nat Commun. 2024 Jun 7;15:4719. doi: 10.1038/s41467-024-48814-z (PMC11161623; doi:10.1038/s41467-024-48814-z)
Supplement: Supplementary file 1 — Supplementary Information [file 41467_2024_48814_MOESM1_ESM.pdf]

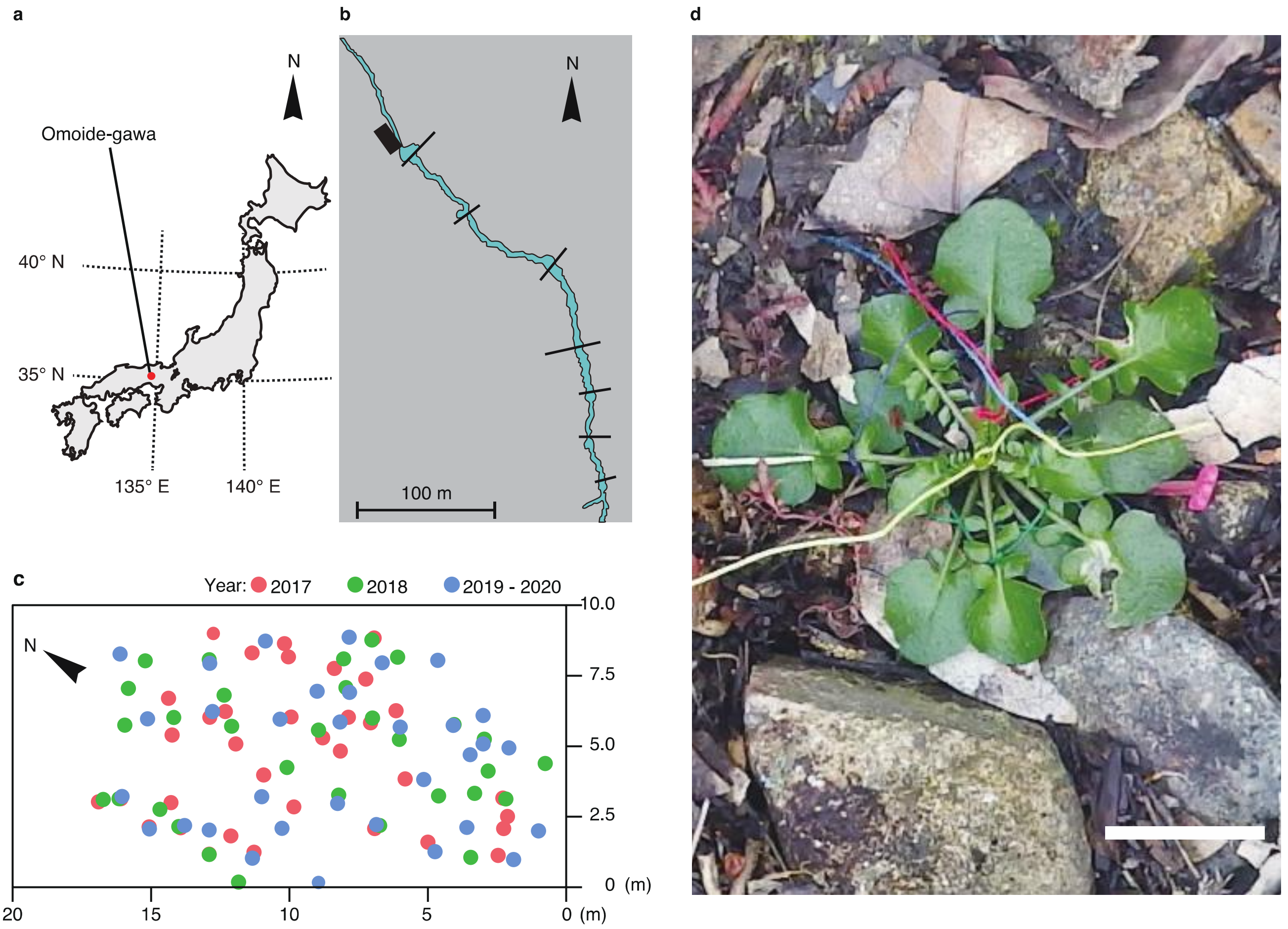

Supplementary Fig. 1: Map showing the locations of the Omoide-gawa study site (a), the main study plot (b) and the plants studied (c), and a photograph showing a plant tagged with coloured threads (d). In **b**, the locations of the study plots are indicated by a closed square. A stream running from top to bottom is shown by blue shading, and thick lines crossing the stream represent erosion control dams. In **c**, the positions of tagged plants are shown for those observed in the year 2017 (red, October 2017 to September 2018), 2018 (green, October 2018 to September 2019), and 2019-2020 (blue, October 2019 to January 2022). In **d**, the scale bar is 20 mm.

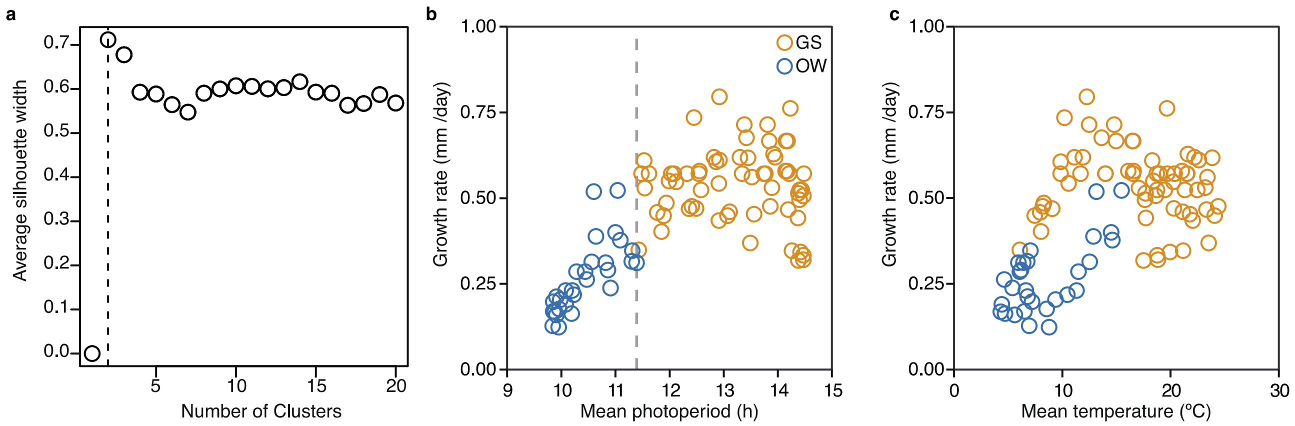

Supplementary Fig. 2: Estimation of the number of clusters in k-mean clustering (a) and leaf growth rate of GS (orange) and OW (blue) cohorts plotted against photoperiod (b) and temperature (c). In **a**, the optimal number of clusters in the *k*-means clustering separating the GS and OW cohorts were estimated using the silhouette methods. The dotted line indicates the optimal number of clusters. In **b**, the grey dotted line indicates the 11.4 h photoperiodic threshold separating the GS and OW cohorts.

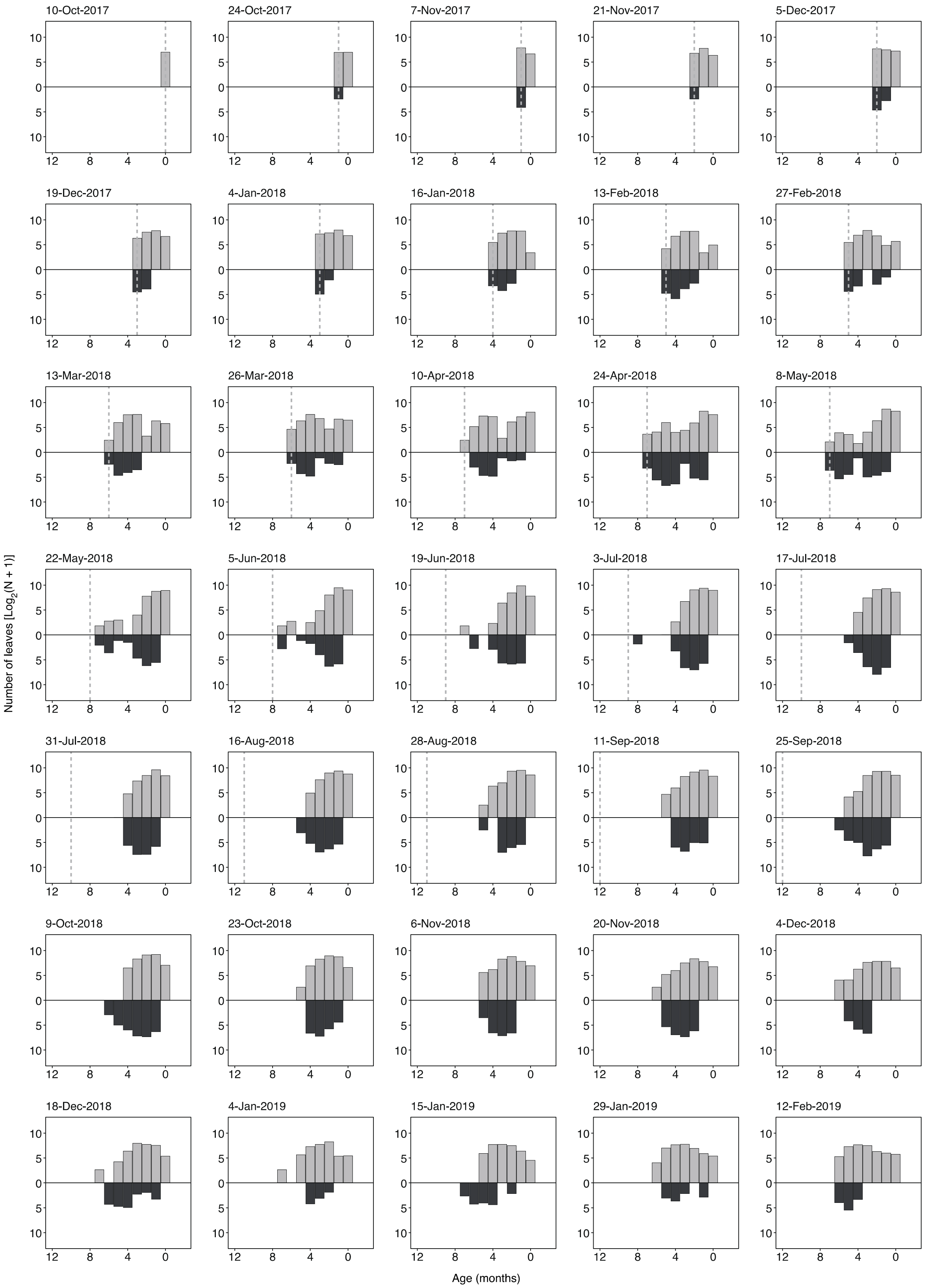

Supplementary Fig. 3: Age structures of extant (grey) and withered (black) leaves (Log$_2$ [number of leaves + 1]) on all biweekly census dates. In the early stages of observation, leaves older than the first tagged cohort (indicated by the vertical dashed lines) do not appear in the plots.

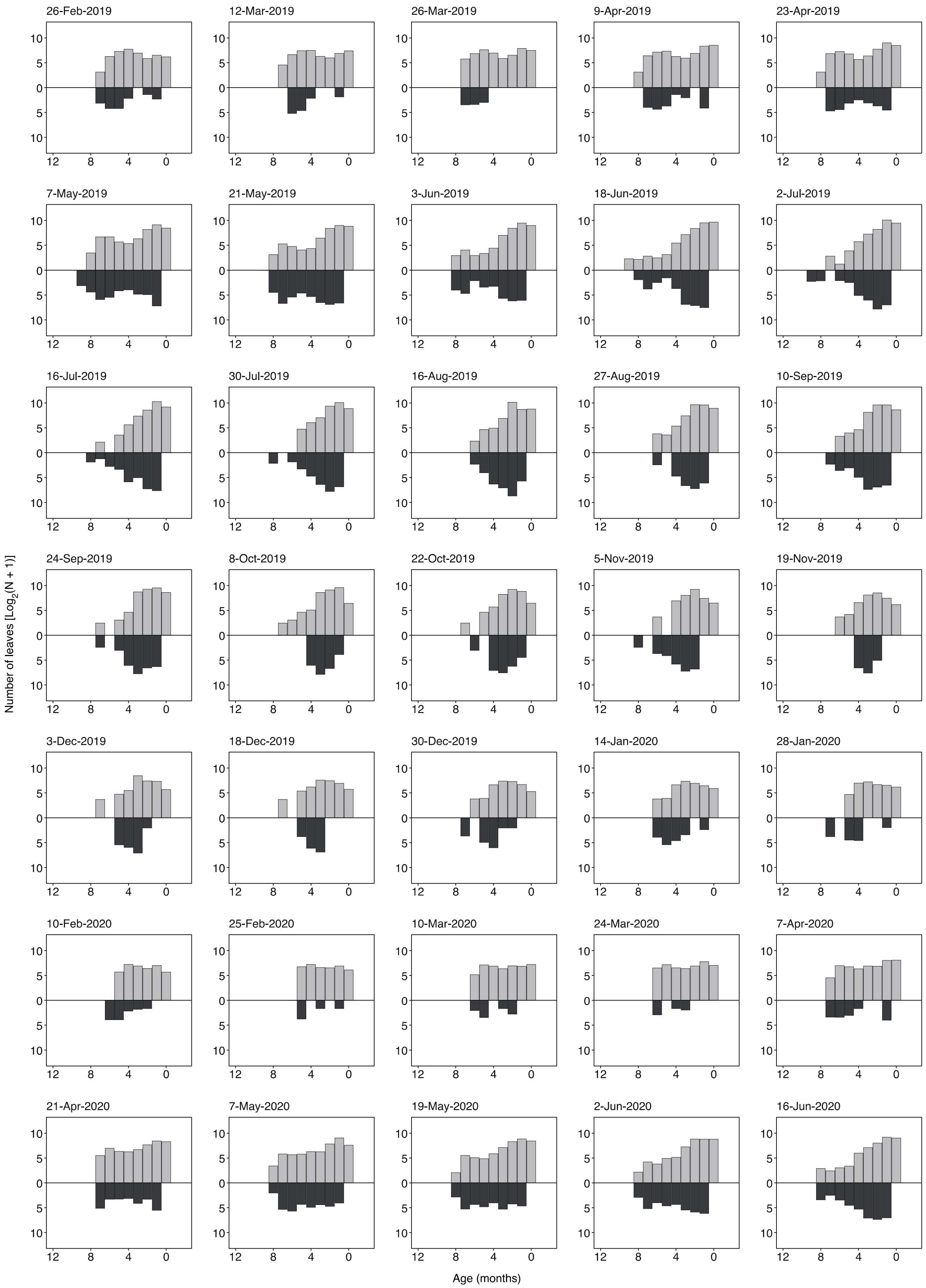

Supplementary Fig. 3: Continued.

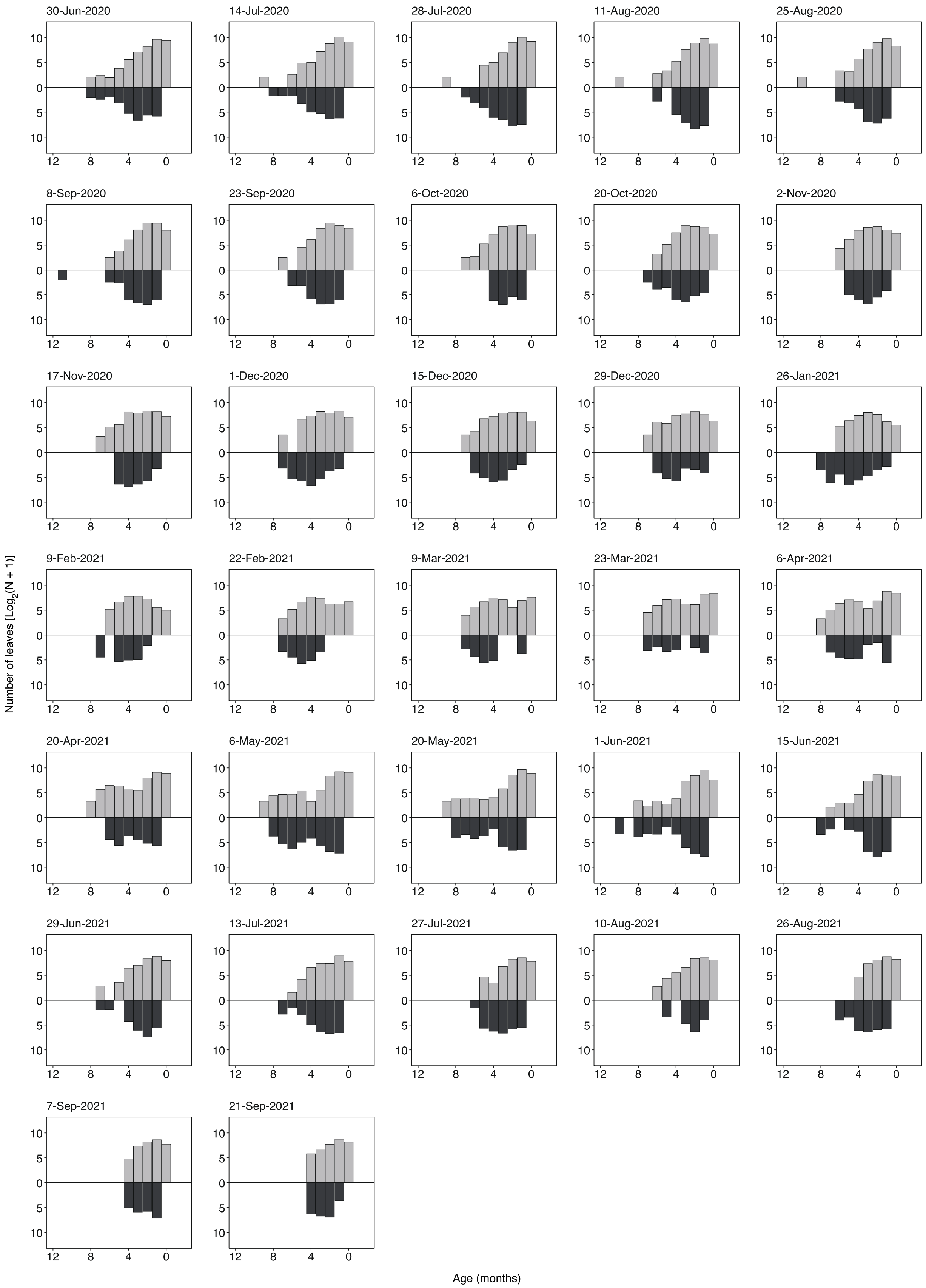

Supplementary Fig. 3: Continued.

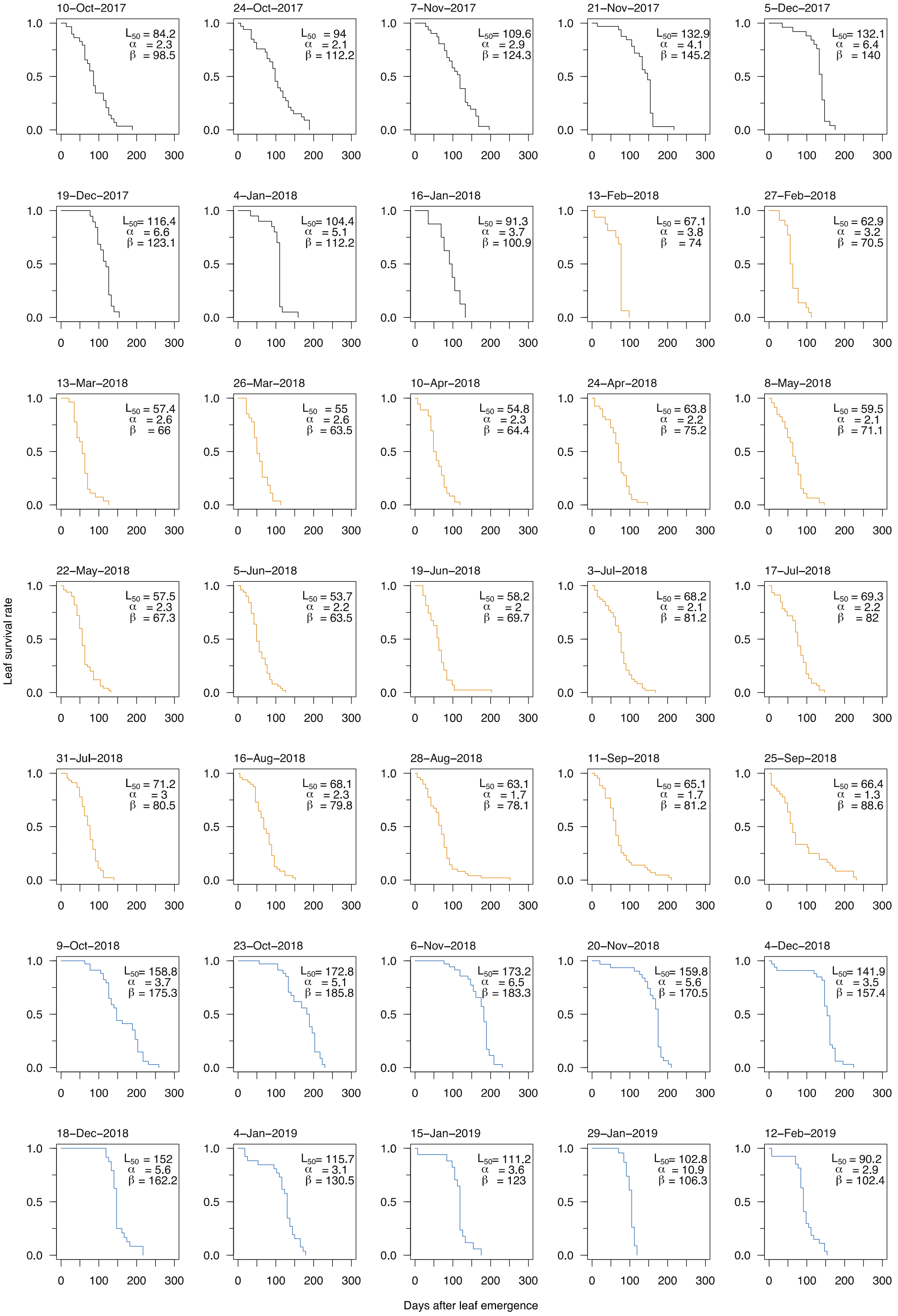
10–Oct–2017
L50 = 84.2 α = 2.3 β = 98.5
24–Oct–2017
L50 = 94 α = 2.1 β = 112.2
7–Nov–2017
L50 = 109.6 α = 2.9 β = 124.3
21–Nov–2017
L50 = 132.9 α = 4.1 β = 145.2
5–Dec–2017
L50 = 132.1 α = 6.4 β = 140
19–Dec–2017
L50 = 116.4 α = 6.6 β = 123.1
4–Jan–2018
L50 = 104.4 α = 5.1 β = 112.2
16–Jan–2018
L50 = 91.3 α = 3.7 β = 100.9
13–Feb–2018
L50 = 67.1 α = 3.8 β = 74
27–Feb–2018
L50 = 62.9 α = 3.2 β = 70.5
13–Mar–2018
L50 = 57.4 α = 2.6 β = 66
26–Mar–2018
L50 = 55 α = 2.6 β = 63.5
10–Apr–2018
L50 = 54.8 α = 2.3 β = 64.4
24–Apr–2018
L50 = 63.8 α = 2.2 β = 75.2
8–May–2018
L50 = 59.5 α = 2.1 β = 71.1
22–May–2018
L50 = 57.5 α = 2.3 β = 67.3
5–Jun–2018
L50 = 53.7 α = 2.2 β = 63.5
19–Jun–2018
L50 = 58.2 α = 2 β = 69.7
3–Jul–2018
L50 = 68.2 α = 2.1 β = 81.2
17–Jul–2018
L50 = 69.3 α = 2.2 β = 82
31–Jul–2018
L50 = 71.2 α = 3 β = 80.5
16–Aug–2018
L50 = 68.1 α = 2.3 β = 79.8
28–Aug–2018
L50 = 63.1 α = 1.7 β = 78.1
11–Sep–2018
L50 = 65.1 α = 1.7 β = 81.2
25–Sep–2018
L50 = 66.4 α = 1.3 β = 88.6
9–Oct–2018
L50 = 158.8 α = 3.7 β = 175.3
23–Oct–2018
L50 = 172.8 α = 5.1 β = 185.8
6–Nov–2018
L50 = 173.2 α = 6.5 β = 183.3
20–Nov–2018
L50 = 159.8 α = 5.6 β = 170.5
4–Dec–2018
L50 = 141.9 α = 3.5 β = 157.4
18–Dec–2018
L50 = 152 α = 5.6 β = 162.2
4–Jan–2019
L50 = 115.7 α = 3.1 β = 130.5
15–Jan–2019
L50 = 111.2 α = 3.6 β = 123
29–Jan–2019
L50 = 102.8 α = 10.9 β = 106.3
12–Feb–2019
L50 = 90.2 α = 2.9 β = 102.4
Leaf survival rate
Days after leaf emergence

23

Supplementary Fig. 4: Kaplan–Meier survival plot for 102 biweekly cohorts. L_{50}, shape (α), and scale (β) parameters are estimated from the survival curve fitting. The GS and OW cohorts are represented by orange and blue plots, respectively. The first eight cohorts (black) were excluded from the k-mean clustering due to lack of growth rate data.

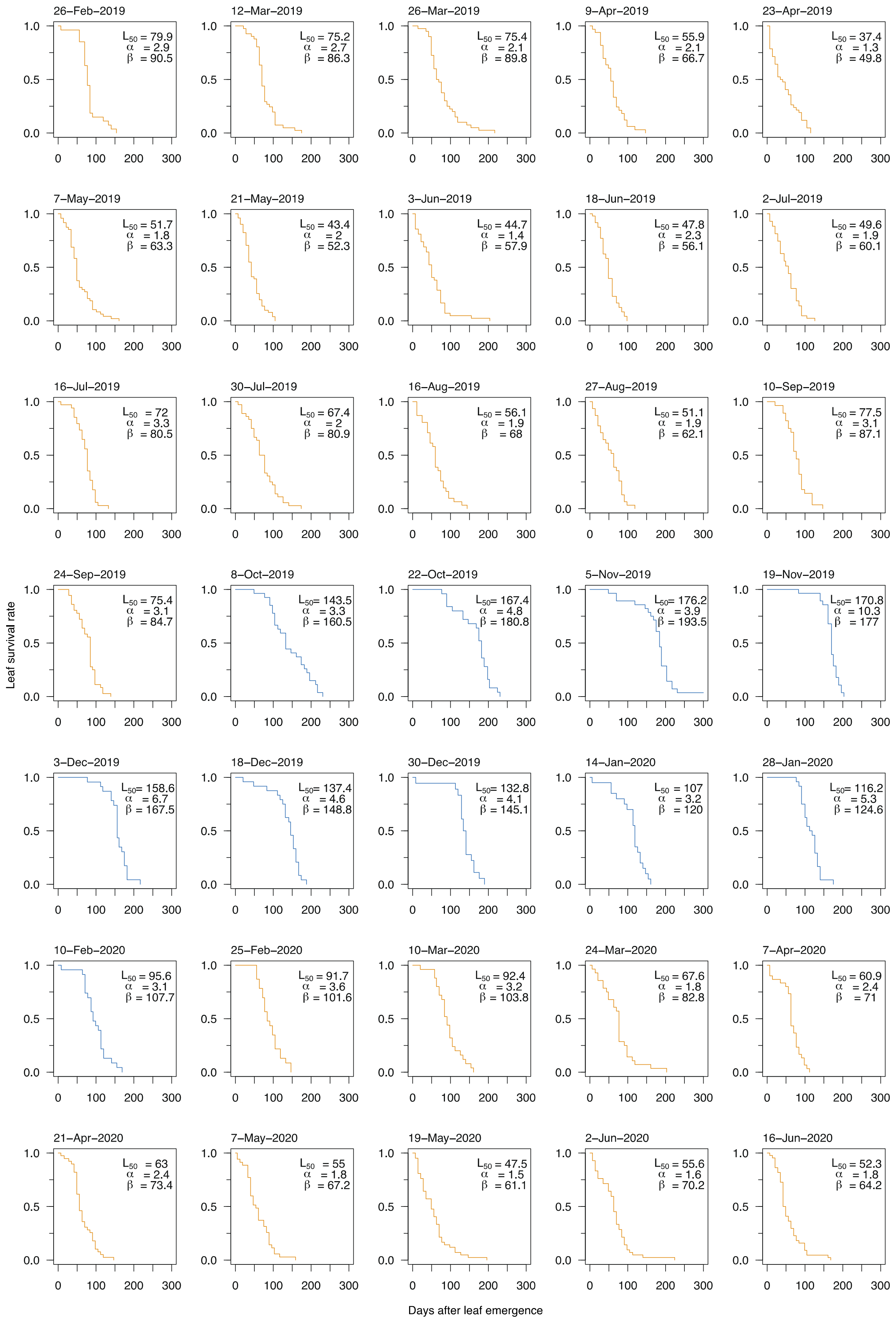

Supplementary Fig. 4: Continued.

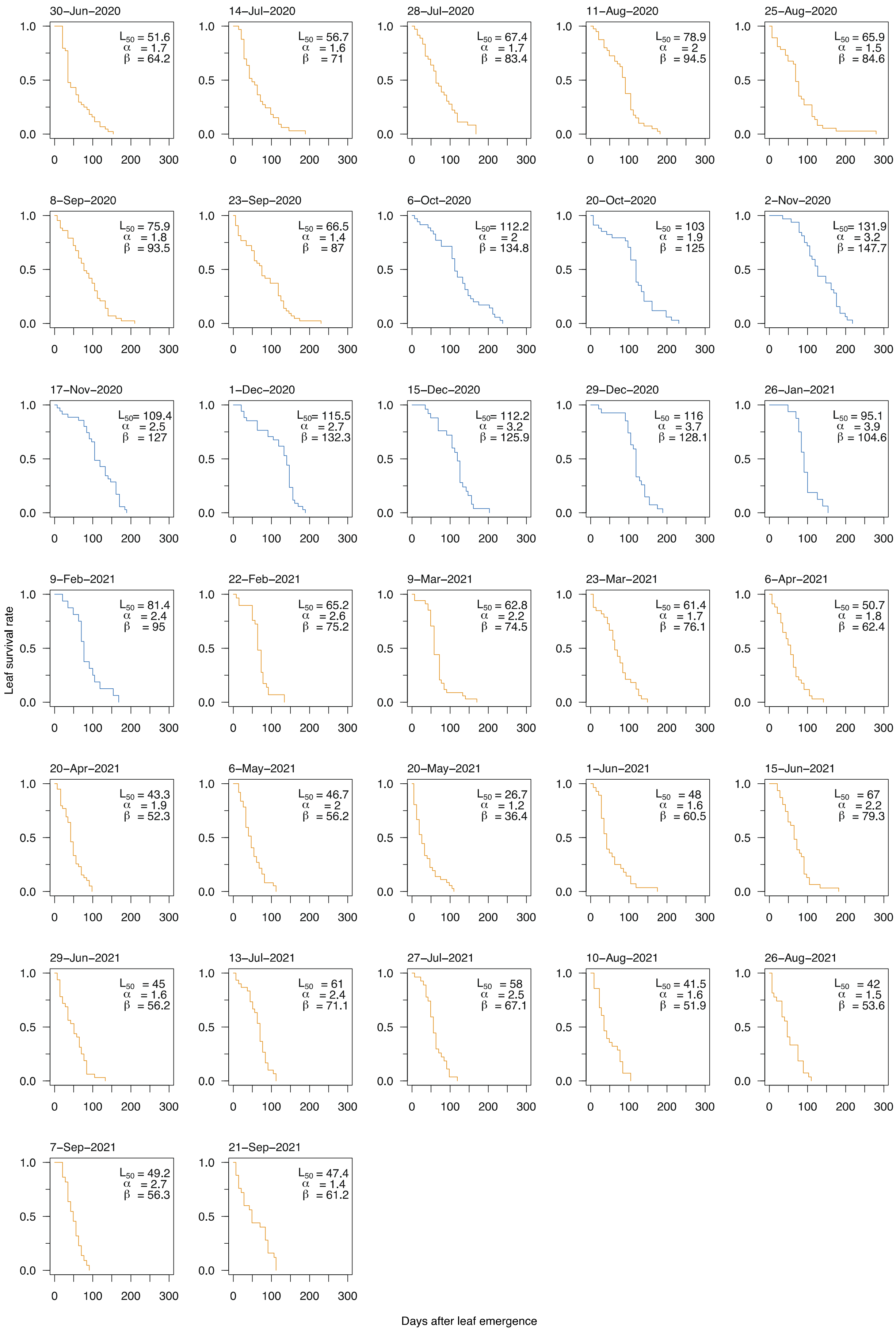

Supplementary Fig. 4: Continued.

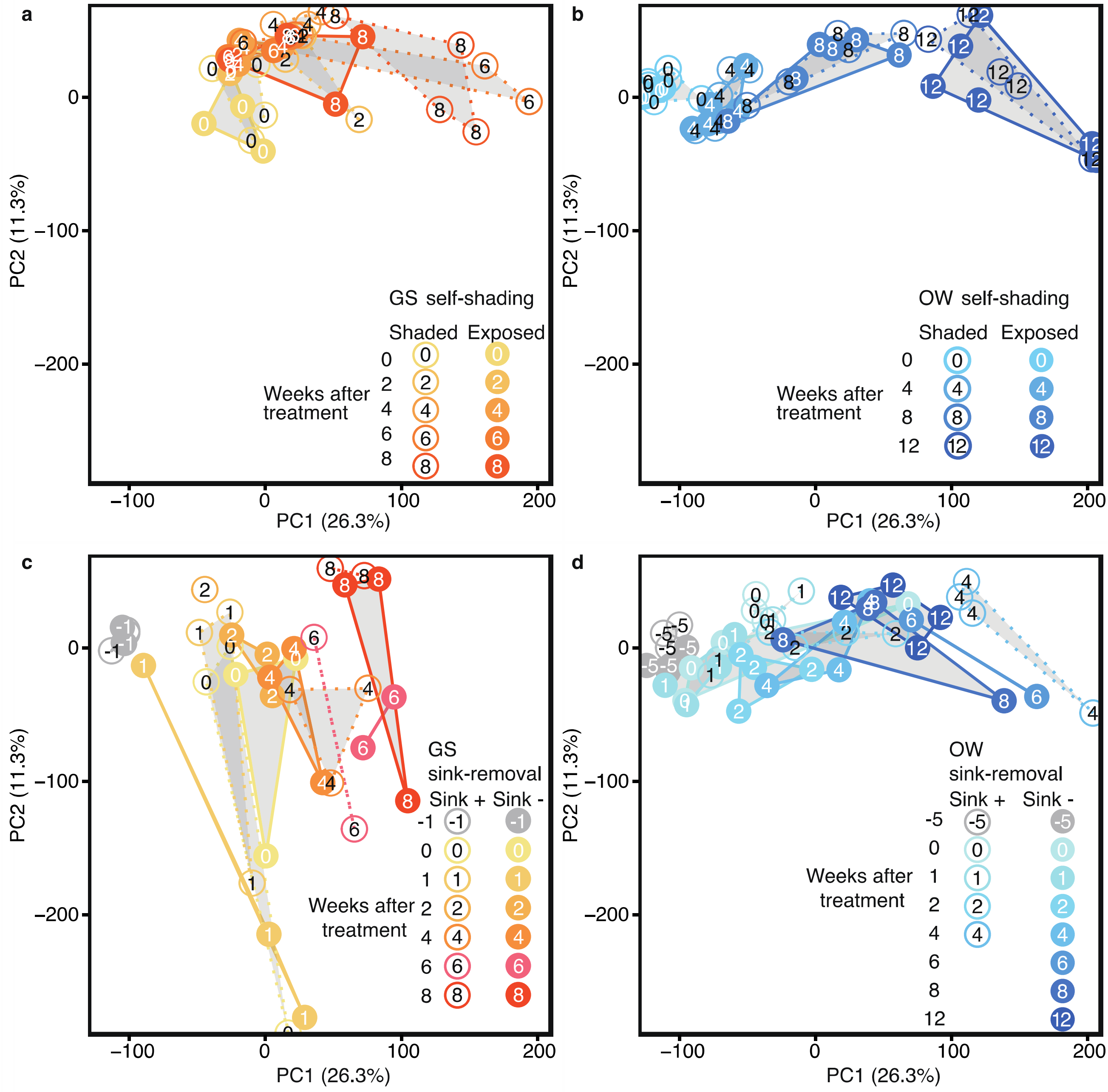

Supplementary Fig. 5: PCA of the samples used for RNA-seq based on all expressed genes (19,292 genes). All samples from the four sets of the experiments were analysed in a single PCA and then plotted separately: **a** GS self-shading; **b** OW self-shading; **c** GS sink-removal; and **d** OW sink-removal. The open and closed circles indicate senescence-enhanced (shaded/sink+) and senescence-repressed (exposed/sink-) treatments, respectively. Replicates were connected by lines (dotted and solid lines for senescence-enhanced and delayed treatments, respectively) and enclosed areas are shown by shade. Numbers in the symbols indicate weeks after treatment initiation.

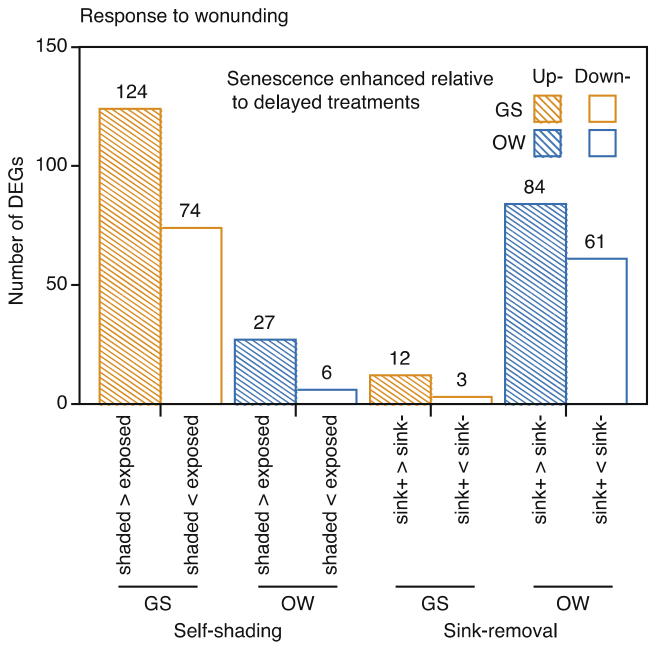

Supplementary Fig. 6: Number of DEGs with the GO ‘response to wounding’ detected between treatments in the four manipulation experiments. Numbers of DEGs are listed on bars.

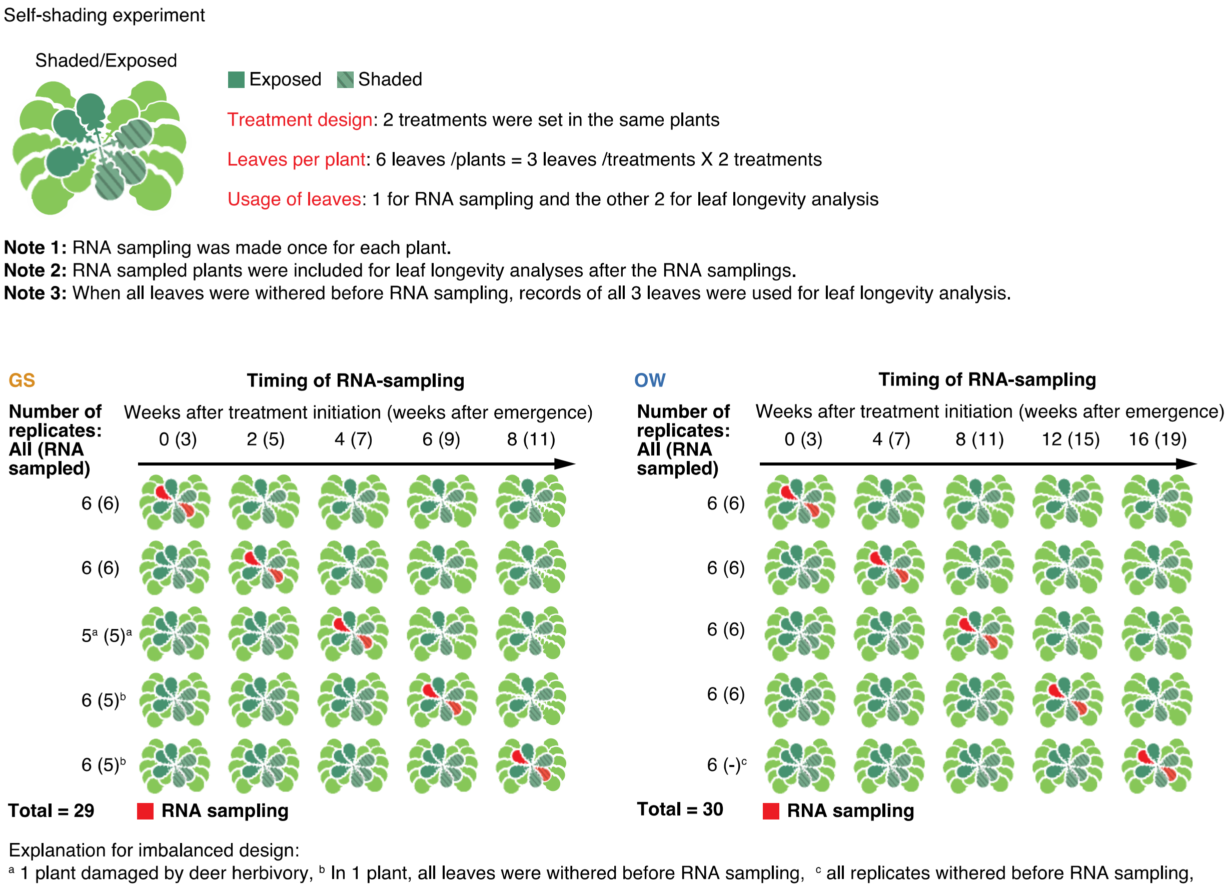

Supplementary Fig. 7: Sampling design for self-shading experiments. Shaded and exposed treatments were conducted within the same individual plant. For each treatment, one leaf was tagged for RNA sampling (red) and the other two for leaf longevity measurements, thus, each individual contains six tagged leaves. Initially, 30 individuals (6 replicates × 5 time points) were tagged for each GS and OW cohort. For each tagged individual, RNA sampling was performed only once after the treatment initiation (0, 2, 4, 6, and 8 weeks for GS, and 0, 4, 8, and 12 weeks for OW). The actual number of individuals for which leaf longevity measurements (for RNA sampling) are listed is in the left row of the bottom diagrams.

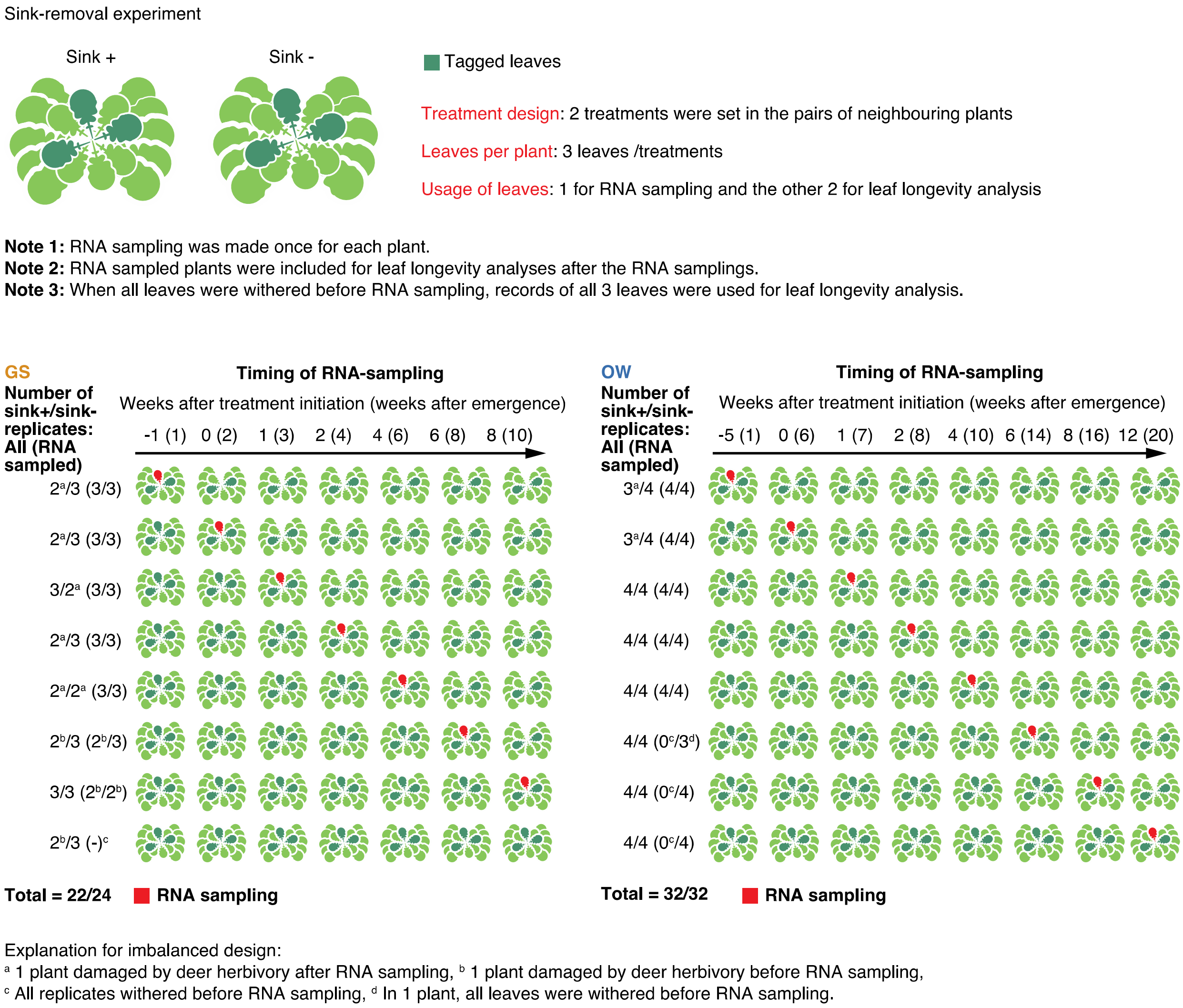

Supplementary Fig. 8: Sampling design for sink-removal experiments. The sink+ and sink-treatments were conducted on a pair of different neighbouring individuals. For each treatment, one leaf is tagged for RNA sampling (red) and the other two for leaf longevity measurements, thus, each individual contains three tagged leaves. Initially, 24 (3 replicates × 8 time points) and 32 (4 replicates × 8 time points) pairs of plants were tagged for the GS and OW cohorts, respectively. For each tagged pair, RNA sampling was performed only once after treatment initiation (-1, 0, 1, 2, 4, 6, 8, and 12 [unsampled] weeks for GS and -5, 0, 1, 2, 4, 6, 8, and 12 weeks for OW). The actual number of individuals for which leaf longevity measurements (for RNA sampling) are listed is in the left row of the bottom diagrams.
