# Supplementary material for: Seasonal switching of integrated leaf senescence controls in an evergreen perennial Arabidopsis
Source: Nat Commun. 2024 Jun 7;15:4719. doi: 10.1038/s41467-024-48814-z (PMC11161623; doi:10.1038/s41467-024-48814-z)
Supplement: Supplementary file 3 — Description of additional supplementary files [file 41467_2024_48814_MOESM3_ESM.pdf]

Description of Additional Supplementary Files

Supplementary Data 1 | List of parameters for all leaf cohorts in the leaf phenology study (Figs 1 and 2).

Supplementary Data 2 | List of parameters for all cohorts used in the manipulation experiments (Figs 3 and 4). L50, L75, and L90 represent days to 50%, 75%, and 90% leaf mortality, respectively.

Supplementary Data 3 | List of senescence-related genes and their classification into the nine senescence gene categories (1, relevant; 0, no information). Whether they are detected as differently expressed genes (DEGs) in the four manipulation experiments is also listed (1, upregulated; -1, downregulated, 0; no DEG or unexpressed).

Supplementary Data 4a | Results of edgeR and gene expression data (Log2 [rpm + 1]) for all detected genes in the GS self-shading experiment. We listed the FDR and logFC values between the shaded and exposed treatments at all sampling times (0, 2, 4, 6, and 8 weeks after treatment initiation).

Supplementary Data 4b | Results of edgeR and gene expression data (Log2 [rpm + 1]) for leaf-senescence-related genes in the GS self-shading experiment. We listed the FDR and logFC values between the shaded and exposed treatments at all sampling times (0, 2, 4, 6, and 8 weeks after treatment initiation).

Supplementary Data 4c | Results of edgeR and gene expression data (Log2 [rpm + 1]) for all detected genes in the OW self-shading experiment. We listed the FDR and logFC values between the shaded and exposed treatments at all sampling times (0, 4, 8, and 12 weeks after treatment initiation).

Supplementary Data 4d | Results of edgeR and gene expression data (Log2 [rpm + 1]) for leaf-senescence-related genes in the OW self-shading experiment. We listed the FDR and logFC values between the shaded and exposed treatments at all sampling times (0, 4, 8, and 12 weeks after treatment initiation).

Supplementary Data 5a | Enriched gene ontology (GO) categories for upregulated shade genes in the GS self-shading experiments. We listed GOID, highest-level GO terms (BP, CC, and MF for biological processes, cellular components, and molecular functions, respectively), GO terms, number of genes in the reference, number of DEGs, fold enrichment, and FDR.

Supplementary Data 5b | Enriched gene ontology (GO) categories for downregulated shade genes in the GS self-shading experiments. We listed GOID, highest-level GO terms (BP, CC, and MF for biological processes, cellular components, and molecular functions, respectively), GO terms, number of genes in the reference, number of DEGs, fold enrichment, and FDR.

Supplementary Data 5c | Enriched gene ontology (GO) categories for upregulated shade genes in the OW self-shading experiments. We listed GOID, highest-level GO terms (BP, CC, and MF for biological processes, cellular components, and molecular functions, respectively), GO terms, number of genes in the reference, number of DEGs, fold enrichment, and FDR.

Supplementary Data 5d | Enriched gene ontology (GO) categories for downregulated shade genes in the OW self-shading experiments. We listed GOID, highest-level GO terms (BP, CC, and MF for biological processes, cellular components, and molecular functions, respectively), GO terms, number of genes in the reference, number of DEGs, fold enrichment, and FDR.

Supplementary Data 6a | Results of edgeR and gene expression data (Log2 [rpm + 1]) for all detected genes in the GS sink-removal experiment. We listed the FDR and logFC values between the sink+ and sink-treatments at all sampling times (-1, 0, 1, 2, 4, 6, and 8 weeks after treatment initiation).

Supplementary Data 6b | Results of edgeR and gene expression data (Log2 [rpm + 1]) for leaf-senescence-related genes in the GS sink-removal experiment. We listed the FDR and logFC values between the sink+ and sink-treatments at all sampling times (-1, 0, 1, 2, 4, 6, and 8 weeks after treatment initiation).

Supplementary Data 6c | Results of edgeR and gene expression data (Log2 [rpm + 1]) for all detected genes in the OW sink-removal experiment. We listed the FDR and logFC values between the sink+ and sink-treatments at all sampling times (-5, 0, 1, 2, 4, 6, 8, and 12 weeks after treatment initiation).

Supplementary Data 6d | Results of edgeR and gene expression data (Log2 [rpm + 1]) for leaf-senescence-related genes in the OW sink-removal experiment. We listed the FDR and logFC values between the sink+ and sink-treatments at all sampling times (-5, 0, 1, 2, 4, 6, 8, and 12 weeks after treatment initiation).

Supplementary Data 7a | Gene ontology (GO) categories for sink+ upregulated genes in the GS sink-removal experiments. We listed GOID, the highest-level GO terms (BP, CC, and MF for biological processes, cellular components, and molecular functions, respectively). GO terms, number of genes in the reference, number of DEGs, fold enrichment, and FDR.

Supplementary Data 7b | Enriched gene ontology (GO) categories for sink+ downregulated genes in the GS sink-removal experiments. We listed GOID, the highest-level GO terms (BP, CC, and MF for biological processes, cellular components, and molecular functions, respectively). GO terms, number of genes in the reference, number of DEGs, fold enrichment, and FDR.

Supplementary Data 7c | Enriched gene ontology (GO) categories for sink+ upregulated genes in the OW sink-removal experiments. We listed GOID, the highest-level GO terms (BP, CC, and MF for biological processes, cellular components, and molecular functions, respectively). GO terms, number of genes in the reference, number of DEGs, fold enrichment, and FDR.

Supplementary Data 7d | Enriched gene ontology (GO) categories for sink+ downregulated genes in the OW sink-removal experiments. We listed GOID, the highest-level GO terms (BP, CC, and MF for

biological processes, cellular components, and molecular functions, respectively). GO terms, number of genes in the reference, number of DEGs, fold enrichment, and FDR.

Supplementary Data 8a | Enriched gene ontology (GO) categories for GS-shaded specific upregulated genes in the comparison between GS self-shading and OW sink-removal experiments. We listed GOID, highest-level GO terms (BP, CC, and MF for biological processes, cellular components, and molecular functions, respectively), GO terms, number of genes in the reference, number of DEGs, fold enrichment, and FDR.

Supplementary Data 8b | Enriched gene ontology (GO) categories for OW-sink+ specific upregulated genes in the comparison between GS self-shading and OW sink-removal experiments. We listed GOID, highest-level GO terms (BP, CC, and MF for biological processes, cellular components, and molecular functions, respectively), GO terms, number of genes in the reference, number of DEGs, fold enrichment, and FDR.

Supplementary Data 8c | Enriched gene ontology (GO) categories for GS shaded and OW sink+ upregulated genes in the comparison between GS self-shading and OW sink-removal experiments. We listed GOID, highest-level GO terms (BP, CC, and MF for biological processes, cellular components, and molecular functions, respectively), GO terms, number of genes in the reference, number of DEGs, fold enrichment, and FDR.

Supplementary Data 8d | Enriched gene ontology (GO) categories for GS-shaded specific downregulated genes in the comparison between GS self-shading and OW sink-removal experiments. We listed GOID, highest-level GO terms (BP, CC, and MF for biological processes, cellular components, and molecular functions, respectively), GO terms, number of genes in the reference, number of DEGs, fold enrichment, and FDR.

Supplementary Data 8e | Enriched gene ontology (GO) categories for OW-sink+ specific downregulated genes in the comparison between GS self-shading and OW sink-removal experiments. We listed GOID, highest-level GO terms (BP, CC, and MF for biological processes, cellular components, and molecular functions, respectively), GO terms, number of genes in the reference, number of DEGs, fold enrichment, and FDR.

Supplementary Data 8f | Enriched gene ontology (GO) categories GS shaded and OW sink+ downregulated genes in the comparison between GS self-shading and OW sink-removal experiments. We listed GOID, highest-level GO terms (BP, CC, and MF for biological processes, cellular components, and molecular functions, respectively), GO terms, number of genes in the reference, number of DEGs, fold enrichment, and FDR.

Supplementary Data 9a | Results of edgeR and gene expression data (Log2 [rpm + 1]) for all detected genes in the GS and OW sink+. We listed the FDR and logFC values between the GS and OW cohorts at four time points (1, 6, 8, and 10 weeks after leaf emergence).

Supplementary Data 9b | Results of edgeR, gene expression data (Log2 [rpm + 1]), and clustering analysis for leaf-senescence-related genes in the GS and OW sink+. We listed the FDR and logFC values between the GS and OW cohorts at four time points (1, 6, 8, and 10 weeks after leaf emergence).

Supplementary Data 10a | Enriched gene ontology (GO) categories for upregulated genes in the GS cohort in the comparison between the GS and OW sink + treatments. We listed GOID, highest-level GO terms (BP, CC, and MF for biological processes, cellular components, and molecular functions, respectively), GO terms, number of genes in the reference, number of DEGs, fold enrichment, and FDR.

Supplementary Data 10b | Enriched gene ontology (GO) categories for upregulated genes in the OW cohort in the comparison between the GS and OW sink + treatments. We listed GOID, highest-level GO terms (BP, CC, and MF for biological processes, cellular components, and molecular functions, respectively), GO terms, number of genes in the reference, number of DEGs, fold enrichment, and FDR.
