# Supplementary material for: Seasonal switching of integrated leaf senescence controls in an evergreen perennial Arabidopsis
Source: Nat Commun. 2024 Jun 7;15:4719. doi: 10.1038/s41467-024-48814-z (PMC11161623; doi:10.1038/s41467-024-48814-z)
Supplement: Supplementary file 14 — Reporting Summary [file 41467_2024_48814_MOESM14_ESM.pdf]

nature portfolio

Corresponding author(s): Hiroshi Kudoh, Genki Yumoto

Last updated by author(s): Apr 18, 2024

# Reporting Summary

Nature Portfolio wishes to improve the reproducibility of the work that we publish. This form provides structure for consistency and transparency in reporting. For further information on Nature Portfolio policies, see our Editorial Policies and the Editorial Policy Checklist.

## Statistics

For all statistical analyses, confirm that the following items are present in the figure legend, table legend, main text, or Methods section.

| n/a | Confirmed | |
|---|---|---|
| ☐ | ☒ | The exact sample size ($n$) for each experimental group/condition, given as a discrete number and unit of measurement |
| ☐ | ☒ | A statement on whether measurements were taken from distinct samples or whether the same sample was measured repeatedly |
| ☐ | ☒ | The statistical test(s) used AND whether they are one- or two-sided<br>*Only common tests should be described solely by name; describe more complex techniques in the Methods section.* |
| ☐ | ☒ | A description of all covariates tested |
| ☐ | ☒ | A description of any assumptions or corrections, such as tests of normality and adjustment for multiple comparisons |
| ☐ | ☒ | A full description of the statistical parameters including central tendency (e.g. means) or other basic estimates (e.g. regression coefficient) AND variation (e.g. standard deviation) or associated estimates of uncertainty (e.g. confidence intervals) |
| ☐ | ☒ | For null hypothesis testing, the test statistic (e.g. $F$, $t$, $r$) with confidence intervals, effect sizes, degrees of freedom and $P$ value noted<br>*Give P values as exact values whenever suitable.* |
| ☒ | ☐ | For Bayesian analysis, information on the choice of priors and Markov chain Monte Carlo settings |
| ☒ | ☐ | For hierarchical and complex designs, identification of the appropriate level for tests and full reporting of outcomes |
| ☐ | ☒ | Estimates of effect sizes (e.g. Cohen's $d$, Pearson's $r$), indicating how they were calculated |

*Our web collection on statistics for biologists contains articles on many of the points above.*

## Software and code

Policy information about availability of computer code

| | |
|---|---|
| Data collection | HiSeq Control Software (v2.2.68), Real Time Analysis (v1.18.66) and BCL2FASTQ (v1.8.4) were used to obtain RNA-seq data. |
| Data analysis | Trimmomatic-0.36, RSEM 1.2.31 and Bowtie2 2.2.9 were used for RNA-seq data processing. R 4.0.2 and Microsoft Excel for Mac 16.16.23 were used for statistical analysis and graph generation. Statistical tests were performed using 'edgeR', 'survminer', 'cluster', 'C50', 'genefilter' and 'exactRankTests' in the R package. We described in the code availability section that the R codes used in this study are deposited at http://sohi.ecology.kyoto-u.ac.jp/AhgRNAseq/Data.zip. |

For manuscripts utilizing custom algorithms or software that are central to the research but not yet described in published literature, software must be made available to editors and reviewers. We strongly encourage code deposition in a community repository (e.g. GitHub). See the Nature Portfolio guidelines for submitting code & software for further information.

## Data

Policy information about availability of data

All manuscripts must include a data availability statement. This statement should provide the following information, where applicable:

- Accession codes, unique identifiers, or web links for publicly available datasets
- A description of any restrictions on data availability
- For clinical datasets or third party data, please ensure that the statement adheres to our policy

The raw RNA-seq reads have been deposited in the DNA Data Bank of Japan (DDBJ) under accession number DRA013140.

# Research involving human participants, their data, or biological material

Policy information about studies with human participants or human data. See also policy information about sex, gender (identity/presentation), and sexual orientation and race, ethnicity and racism.

| | |
|---|---|
| Reporting on sex and gender | N/A |
| Reporting on race, ethnicity, or other socially relevant groupings | N/A |
| Population characteristics | N/A |
| Recruitment | N/A |
| Ethics oversight | N/A |

Note that full information on the approval of the study protocol must also be provided in the manuscript.

# Field-specific reporting

Please select the one below that is the best fit for your research. If you are not sure, read the appropriate sections before making your selection.

☐ Life sciences ☐ Behavioural & social sciences ☒ Ecological, evolutionary & environmental sciences

For a reference copy of the document with all sections, see nature.com/documents/nr-reporting-summary-flat.pdf

# Ecological, evolutionary & environmental sciences study design

All studies must disclose on these points even when the disclosure is negative.

**Study description**

We conducted a series of field observations and experiments to study the seasonal variation in leaf longevity and its determinants.

1. Four years of biweekly leaf tagging and weekly records and measurements of leaf longevity and growth [a total of 3,334 leaves (102 biweekly cohorts) for 90 individuals (30 /year, in the last two years a common set of 30 individuals was used in the last two years) in a natural population of Arabidopsis halleri subsp. gemmifera.

2. Mathematical modelling (decision tree analyses) using 94 cohorts to estimate environmental determinants of leaf longevity. The environmental factors used were simple moving averages of photoperiod, solar radiation and temperature for 33 days after leaf emergence.

3. Field manipulation experiments, i.e. self-shading and sink removal experiments for the representative growth season (GS) and overwintering (OW) leaf cohorts, to evaluate the effects of local light environments and translocation on leaf longevity. The number of replicates is [GS, N = 58 (exposed), N = 60 (shaded); OW, N = 63 (exposed), N = 60 (shaded)] in self-shading experiments and [GS, N = 39 (sink +), N = 43 (sink -); OW, N = 71 (sink +), N = 65 (sink -)] in sink-removal experiments.

4. Time-series RNA-seq analyses on samples from the field manipulation experiments to identify genes that show expression changes in response to treatments. The number of replicates of the self-shading experiment in GS and in OW is 40 leaves (4 replicates per time point per treatment × 5 time points × 2 treatments) and 40 leaves (5 replicates × 4 time points × 2 treatments), respectively. The number of replicates of the sink-removal experiment in GS and in OW is 38 leaves ([2 replicates at 6,8 weeks after treatment initiation (wat) and 3 replicates at other 5 time points] × 2 treatments) and 48 leaves ([2 replicates at 6 wat, 3 replicates at 0 wat and 4 replicates at other 6 time points] × 2 treatments), respectively. No samples were taken from the sink+ treatment in OW after 6 wat in the latter experiment because all leaves had withered before these time points. All experiments were performed in a counterbalanced design between treatments.

**Research sample**

All studies were conducted in a natural population of the perennial Arabidopsis halleri subsp. gemmifera (A. halleri) in central Japan, and the evergreen habit of the species allowed us to conduct a 4-year study on leaf longevity and leaf dynamics. Arabidopsis halleri belongs to the Brassicaceae family and is closely related to A. thaliana, which allowed us to identify homologous genes in the RNA-seq analyses.

For studies 1 and 2 (numbers correspond to those in the study description), 30 in-field monitoring plants were selected during the 4-year field survey by establishing twelve 3 × 3 m subplots within the main plot where A. halleri was relatively abundant. One to three plants were selected with a distance between plants > 1 m. The selected plants had more than four leaves (average 18 leaves) and no signs of obvious damage.

For studies 3 and 4, paired plants of similar size were selected from naturally growing A. halleri in the same population. See study description for replicates.

**Sampling strategy**

For studies 1 and 2, we used a sample size of 30 individuals for long-term leaf phenology, which was the maximum number that could be surveyed after arrival at the study site and before sunset, especially in winter. A maximum of two leaves per individual were

tagged biweekly for each cohort, and the average number of leaves per cohort was 32.7. The biweekly observations were repeated over a four-year period to confirm that we observed similar patterns in the statistics used in this study in different years. We therefore considered the sample sizes to be sufficient.

For study 3, the difference in the time series of leaf longevity between the experimental treatments was compared using the log-rank test (a time-stratified Cochran-Mantel-Haenszel test) using R version 4.0.2. In the online manual of the corresponding package, the time-series analyses were applied for the 12 vs 14 sample case. The sample size in this study was 39-71 per treatment. Judging from the probability levels, the sample size was sufficient to detect the difference in L50.

For study 4, the number of replicates/time point/treatment was at least two or more in each manipulation experiment. We judged that two replicates per treatment was sufficient as long as we were arguing for genes that were designated as DEG under a certain level of FDR. As RNA-seq analyses are expensive, two replicates per treatment was also a compromise between statistical requirements and research affordability.

Data collection

Data collection was described in detail in the Methods section. Plant material was sampled by the two permanent observers, G.Y. and J.S. One of them, G.Y., recorded the presence and absence of leaves and measured leaf length with a ruler.

Timing and spatial scale

All measurements were conducted on a 20 × 25 m rectangular plot in a natural population of Arabidopsis halleri at the Omoide-gawa study site in Hyogo Prefecture, with approximately 300 individual plants in the plot.

For studies 1 and 2, all measurements were taken from one population (Omoide-gawa, Hyogo, Japan) at regular intervals from 10 October 2017 to 11 January 2022 (we tagged leaves every two weeks for three years and observed their fate every week until the last leaf died). Since the average leaf longevity of the target species was 83 days (maximum 308 days), the weekly survey was sufficient to record the variation in leaf longevity.

For study 3, all measurements were made weekly from 2 July 2019 to 12 November 2019 (self-shading in GS), from 21 July 2020 to 24 November 2020 (sink-removal in GS), from 5 January 2021 to 20 May 2021 (self-shading in OW), and from 8 February 2022 to 5 July 2022 (sink-removal in OW). The timings were chosen to apply the treatments to typical GS and OW cohorts.

For study 4, the self-shading experiments in GS were 23 July, 6 August, 20 August, 3 September and 17 September (3 weeks after tagging on the day of treatment initiation; 2, 4, 6 and 8 wats), in OW were 26 January, 23 February, 23 March and 20 April (3 weeks after tagging on the day of treatment initiation; 4, 8 and 12 wats); 4, 8 and 12 wat), and the sink-removal experiment in GS were 28 July (1 week after tagging before treatment), 4 August (the day of treatment initiation), 11 August, 18 August, 1 September, 15 September and 29 September (1, 2, 4, 6 and 8 wat), in OW were 15 February (1 week after tagging before treatment), 22 March (the day of treatment initiation), 29 March, 5 April, 19 April, 2 May, 17 May and 14 June (1, 2, 4, 6, 8 and 12 wat). The timing of sampling was chosen to cover the whole process of leaf senescence in all treatments.

Data exclusions

In all cases, leaves damaged by deer were excluded from the data analysis of leaf longevity and leaf growth rate.

Reproducibility

For studies 1 and 2, we have four years of data and the results of the analyses were more or less similar, so the attempt to repeat the measurement over four years was successful. For studies 3 and 4, each experiment was conducted once, but at least leaf longevity in the control treatment was observed repeatedly in the leaf phenology study.

Randomization

For 1, the labelled plants have been selected to represent the natural population. Plants were placed on a grid of stones 1 m apart in a 20 × 25 m plot and 200 individuals were selected from those closest to the stones. From the selected individuals, 1-3 individuals are selected in a 3 × 3 m subplot from those that have not been observed to be damaged by feeding and are at least 1 m away, depending on the density of the plants.
For 3 and 4, the individuals used in the self-shading experiments were selected from 1-3 individuals outside the 200 individuals that were also more than 1 m away from the 3 × 3 m subplot. For the individuals used in the sink-removal experiment, two individuals with approximately the same rosette diameter within 1 m were used as a pair, and 1-3 pairs of individuals from the 3 × 3 m subplots were selected from individuals outside the 200 individuals that were similarly more than 1 m apart.
Participants were not randomised.

Blinding

No blinding was used. As this was a long-term field study in a remote natural plant population, the study required a range of skills, local experience and personalised equipment to conduct the study under field conditions for years. Therefore, blinding was not relevant to our study.

Did the study involve field work? ☒ Yes ☐ No

# Field work, collection and transport

Field conditions

Field surveys and material collection took place every Tuesday from October 2017 to January 2022. Data on temperature and solar radiation at this time were collected using data loggers placed at the study site. The data are available with the R codes used for the analyses (see 'Code availability' in the main text). For day length, data for Nishiwaki City, Hyogo Prefecture were obtained from the following URL (https://sunrise.maplogs.com/ja/nishiwaki_hyogo_prefecture_japan.75564.html)

Location

All field surveys and material collection were conducted in a natural population of Arabidopsis halleri, Omoide-gawa (Hyogo prefecture; 35º10'N, 134º93'E, alt. ca. 200 m, Kudoh et al., 2018), Japan.

| | |
|---|---|
| Access & import/export | We have notified the local community of our activities, obtained undocumented permission and have been conducting a series of studies since 2005. The study area and species are not designated as a conservation area or target for protection. |
| Disturbance | In this study, all leaf sampling and experimental treatments (removal of flowering stems and new leaves) were carried out without destroying individual plants. All tags and strings were removed from the field site at the end of the measurements. |

# Reporting for specific materials, systems and methods

We require information from authors about some types of materials, experimental systems and methods used in many studies. Here, indicate whether each material, system or method listed is relevant to your study. If you are not sure if a list item applies to your research, read the appropriate section before selecting a response.

## Materials & experimental systems

| n/a | Involved in the study | |
|---|---|---|
| ☒ | ☐ | Antibodies |
| ☒ | ☐ | Eukaryotic cell lines |
| ☒ | ☐ | Palaeontology and archaeology |
| ☒ | ☐ | Animals and other organisms |
| ☒ | ☐ | Clinical data |
| ☒ | ☐ | Dual use research of concern |
| ☐ | ☒ | Plants |

## Methods

| n/a | Involved in the study | |
|---|---|---|
| ☒ | ☐ | ChIP-seq |
| ☒ | ☐ | Flow cytometry |
| ☒ | ☐ | MRI-based neuroimaging |

# Dual use research of concern

Policy information about dual use research of concern

## Hazards

Could the accidental, deliberate or reckless misuse of agents or technologies generated in the work, or the application of information presented in the manuscript, pose a threat to:

| No | Yes | |
|---|---|---|
| ☒ | ☐ | Public health |
| ☒ | ☐ | National security |
| ☒ | ☐ | Crops and/or livestock |
| ☒ | ☐ | Ecosystems |
| ☒ | ☐ | Any other significant area |

## Experiments of concern

Does the work involve any of these experiments of concern:

| No | Yes | |
|---|---|---|
| ☒ | ☐ | Demonstrate how to render a vaccine ineffective |
| ☒ | ☐ | Confer resistance to therapeutically useful antibiotics or antiviral agents |
| ☒ | ☐ | Enhance the virulence of a pathogen or render a nonpathogen virulent |
| ☒ | ☐ | Increase transmissibility of a pathogen |
| ☒ | ☐ | Alter the host range of a pathogen |
| ☒ | ☐ | Enable evasion of diagnostic/detection modalities |
| ☒ | ☐ | Enable the weaponization of a biological agent or toxin |
| ☒ | ☐ | Any other potentially harmful combination of experiments and agents |
